# Detecting Differential Item Functioning across Multiple Groups Using Group Pairwise Penalty

**DOI:** 10.1017/psy.2025.10034

**Published:** 2025-08-11

**Authors:** Weicong Lyu, Chun Wang, Gongjun Xu

**Affiliations:** 1 Faculty of Education, University of Macau, Macau, China; 2 College of Education, University of Washington, Seattle, WA, USA; 3 Department of Statistics, University of Michigan, Ann Arbor, MI, USA

**Keywords:** regularization, differential item functioning, intersectionality, truncated *L*_1_ penalty

## Abstract

We introduce a novel regularization method for detecting differential item functioning (DIF) in two-parameter logistic (2PL) models. Existing regularization methods require choosing a reference group and using an 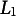 penalty (LP) to shrink the item parameters of focal groups toward those of the reference. This approach has two key limitations: (1) shrinking all focal groups toward a reference is inherently unfair, as results are affected by the choice of reference and direct comparison among focal groups is unavailable and (2) the LP leads to biased estimates because it overly shrinks large nonzero parameters toward zero. These limitations are particularly problematic for intersectional DIF, where various identity aspects intersect to create multiple smaller groups. Our method addresses these issues by penalizing item parameter differences between all pairs of groups using a truncated LP, thereby treating groups equally and avoiding excessive penalization of large differences. Simulations demonstrate that the proposed method outperforms existing approaches by accurately identifying items exhibiting DIF even with multiple small groups. Application to two real-world datasets further illustrates its utility. We recommend this method as a more equitable and precise tool for DIF detection. The proposed method is available as D2PL_pair_em() in the R package VEMIRT (https://map-lab-uw.github.io/VEMIRT).

## 1 Introduction

Differential item functioning (DIF) has long been a significant concern in psychometrics. In simple terms, DIF occurs when individuals with the same ability level respond differently to a particular test item. For instance, a math question might seem easier to a male student but more difficult to a female student, despite both having the same overall math ability. Previous research has demonstrated that DIF is prevalent across various educational and psychological assessments, possibly due to differences in sex, ethnicity, language, culture, and curriculum (Huang et al., 2016; Taylor & Lee, 2011; Teresi et al., 2021; Zenisky et al., 2004).

Addressing DIF is crucial for ensuring measurement accuracy. Psychometric models typically assume that test items function uniformly for all respondents. When this assumption is violated, it leads to biased estimates of respondent and item parameters, rendering subsequent analyses and conclusions questionable (Borsboom et al., 2002; Millsap, 2010). The biased estimates may lead to a significant fairness issue, particularly in high-stakes testing scenarios. If an item disproportionately favors one group of respondents, it artificially inflates their scores, thereby misrepresenting their true abilities and creating an unfair advantage over others (Cole & Zieky, 2001; Zumbo, 2007).

Numerous approaches for detecting DIF have been proposed, many of which are based on item response theory (IRT). When researchers have prior knowledge that certain items are definitely DIF-free, these items can serve as anchors to help calibrate the parameter estimates of other items (Kopf et al., 2015a). However, in practice, such prior knowledge is often unavailable, leading to the development of DIF detection methods that automatically identify anchor items (Chen et al., 2023; Cohen et al., 1996; Kopf et al., 2015b; Lyu et al., 2025; Tutz & Schauberger, 2015; Wang et al., 2023). Among these, the regularization approach is particularly promising. This method involves estimating a multiple-group IRT model while using the 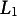 (lasso) penalty (Tibshirani, 1996) or its variants to shrink group differences in item parameters toward zero. Previous studies have demonstrated its effectiveness (Belzak & Bauer, 2020; Lyu et al., 2025; Magis et al., 2015; Schauberger & Mair, 2020; Tutz & Schauberger, 2015; Wang et al., 2023).

Despite its promise, the existing regularization approach has several limitations. Firstly, like lasso regression with dummy variables, it requires researchers to specify a reference group and shrink the differences between each focal group and this reference group toward zero. Researchers often choose a large, advantaged group, such as White males, as the reference because the estimation for larger groups tends to be more accurate, and comparisons between the advantaged group and others are often of interest. However, this approach does not offer a direct comparison between focal groups, such as White females and Black males. Instead, it requires re-estimating the model with one focal group as the new reference. This not only increases computational time but also introduces asymmetry and potential confusion: DIF found for group B when group A is the reference might not appear for group A when group B is the reference. Moreover, selecting one group as the reference is inherently unfair to other groups, as all focal groups are shrunk toward the reference group, disregarding differences among the focal groups themselves.

A trickier and more subtle issue related to unfairness is model identification. When we allow groups to differ in both ability distributions (i.e., impact) and item parameters (i.e., DIF), the IRT model is not identified (Chen et al., 2014). That is, we cannot statistically distinguish between alternative explanations for observed group differences. For instance, the same response pattern could be attributed either to (1) Group A having a much lower mean ability than Group B with no DIF or to (2) the two groups having equal mean ability but all items strongly favoring Group B. Intermediate cases, such as Group A having slightly lower mean ability and all items slightly favoring Group B, are also statistically indistinguishable from these two possibilities. While this is an extreme example, similar identification issues arise in more realistic settings. For example, one cannot statistically distinguish between (1) Group A having a lower mean ability with 40% of the items favoring Group A and (2) the groups having equal mean ability with the remaining 60% of the items favoring Group B. Regularization methods address this problem by automatically identifying anchor items through penalization (Wang et al., 2023), based on the implicit sparsity assumption that most DIF parameters are zero (Chen et al., 2023). In other words, group differences are primarily attributed to impact whenever possible, and only residual differences are attributed to DIF. Again, the automatic selection of anchor items depends on the choice of the reference group because anchor items are chosen by minimizing DIF between focal groups and the reference, while DIF among focal groups is not explicitly taken into account. As a result, existing regularized DIF detection methods, which require a prespecified reference group, lead to a local rather than a global optimum.

Secondly, the 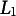 penalty (LP) can produce biased estimates because it shrinks all parameter estimates toward zero, even very large ones (Tibshirani, 1996). To address this, two-step estimation procedures are often used in practice: a first step with the LP for variable selection and then a second debiasing step. Building on this concept, for instance, Wang et al. (2023) extended the expectation–maximization (EM) algorithm for IRT model estimation to the expectation–maximization–maximization (EMM) algorithm. Although EMM has shown good performance in simulation studies, its theoretical performance guarantee has yet to be established.

These limitations are particularly problematic when analyzing a large number of groups, especially when intersectionality is involved. Intersectionality examines how various identity aspects intersect to create multiple smaller groups (also known as social strata), which will further complicate DIF detection (Cole, 2009). Although some existing approaches can handle multiple covariates (i.e., multiple axes of identities), they mostly considered different aspects of identities as additive (Hancock, 2007). This additive approach treats the advantages or disadvantages conferred through simultaneous possession of multiple social positions as simply accumulated, whereas intersectionality theorists posit that inequalities are generated by numerous interlocking systems of privilege and oppression, such as sexism and ageism (Bowleg, 2012). Adding interaction terms helps address intersectionality, but inevitably introduces computation challenges due to complex, saturated models that often require large sample sizes.

Some other existing DIF methods have been adapted for intersectional DIF scenarios. In particular, Russell et al. (2021) applied the standardized D-static method proposed by Dorans & Kulick (1986), which estimates abilities using total scores and compares the percentage of correct responses across groups, both of which can be contaminated by DIF. Their method may also lack statistical power when the sample size is small. Belzak (2023) applied logistic regression with regularization to respondent-level covariates and person ability measured by total scores. While this approach can accommodate nonadditive effects of covariates, the total score is not an ideal proxy for ability because it may be contaminated by DIF. Parker et al. (2024) recently explored intersectional DIF in an introductory computing assessment using the item-focused tree approach proposed by Tutz & Berger (2016). However, the recursive partitioning algorithm is computationally demanding, and as a greedy algorithm, it does not guarantee a globally optimal solution. Other approaches, such as the likelihood ratio test introduced by Thissen et al. (1988), may also be applicable to intersectional DIF detection, although significance testing also often struggles with small sample sizes.

To overcome the limitations of current regularization approaches, this study proposes a new regularization method for DIF detection in the context of the two-parameter logistic (2PL) model, which is among the simplest and most widely used IRT models for dichotomous responses. To address the first limitation, rather than shrinking each focal group toward the reference group, we shrink the *differences* between every pair of groups toward zero, ensuring that no group is designated as a reference or focal group. Instead, all groups are treated equally. To address the second limitation, instead of the commonly used LP, we adopt a truncated LP (TLP) approach, which does not further penalize large differences but remains constant when the difference exceeds a certain threshold. For the estimation, we develop an efficient EM algorithm using the difference convex (DC) programming (Tao & Souad, 1986) and the alternating direction method of multipliers (ADMM; Boyd et al., 2010). Our simulation study demonstrates the clear advantages of the proposed method. The R code implementing the proposed method is provided as the function D2PL_pair_em() in the R package VEMIRT, which is publicly available at https://map-lab-uw.github.io/VEMIRT. The source code for the function can be accessed directly at https://github.com/MAP-LAB-UW/VEMIRT/blob/master/R/D2PL_pair_em.R.

The remainder of this article is organized as follows. First, we present our proposed method in detail. Next, we describe the design of our simulation study and discuss the results. We then apply the proposed method to real-world datasets to demonstrate its practical applicability. Finally, we conclude the article with a discussion of our findings and suggestions for future research.

## 2 Method

### 2.1 Overview

#### 2.1.1 Model setting

Under the original 2PL framework, the probability that respondent *i* answers item *j* correctly is modeled as 

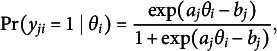

where 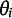 is respondent *i*’s latent trait, and 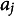 and 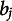 are item *j*’s slope and negative intercept, respectively. Under this setting, all the items function in the same way across all the respondents. When respondents come from *S* social strata or groups, we replace 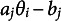 with 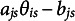 for the *i*th respondent from group *s*. Here, each group is allowed to have its own item parameters, and our goal is to decide whether item parameters are different across groups.

Let *N*, 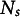 , and *J* denote the total number of respondents, the number of respondents in group *s*, and the total number of items, respectively. The probability that the *i*th respondent of group *s* gives response vector 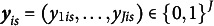 is 
(1)

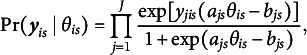

and the method of marginal maximum likelihood estimation maximizes the log marginal likelihood function 
(2)

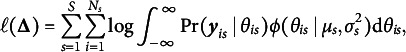

where 

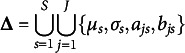

is the set of item and group parameters to estimate, and 
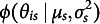
 is the probability density of 
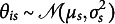
 . Even when there is only one group, the model in (1) is not identified because the metric of the latent variable 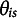 is not determined. The conventional way is to assume that 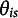 follows a standard normal distribution such that it has zero mean and unit variance (Bock & Aitkin, 1981). In this study, we allow impact to be present, i.e., latent traits of respondents from different groups may have different distributions. One possible way to fix the metric is to let 
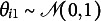
 and freely estimate 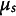 and 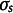 for 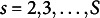 .

To detect item parameter heterogeneity among groups, we impose a penalty over item parameter differences across groups and expect that small differences are shrunk to exactly zero. Existing regularization methods require researchers to select one group as the reference, and all other groups become focal groups. These focal groups are then shrunk toward the reference by penalizing the differences in item parameters between each focal group and the reference. For example, Wang et al. (2023) and Lyu et al. (2025) specified Group 1 as the reference and imposed the LP, i.e., 

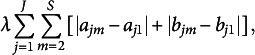

where 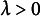 is a tuning parameter that controls the strength of regularization. As discussed in the introduction, this LP approach has several limitations, such as inequity across groups, no direct comparisons among focal groups, asymmetry of DIF detection, and bias caused by overshrinkage of LP.

#### 2.1.2 Regularization with TLP

Ideally, we hope to impose the 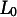 penalty, 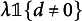, which leads to sparsity by penalizing all nonzero differences equally. However, the 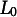 penalty presents computational challenges because it is neither continuous nor convex. As a result, the LP is commonly adopted as a surrogate. Different from the 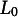 penalty, LP penalizes more heavily when the magnitude of *d* gets greater, which is undesirable and leads to biased estimates due to overshrinkage (Tibshirani, 1996). To solve this problem, it is required that the penalty should work similarly to LP when *d* is close to zero but stay constant when 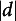 is large. In this study, we propose using the TLP (Shen et al., 2012), 

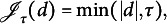

for regularization because its simple structure leads to a relatively simple optimization algorithm.

**Figure 1:**
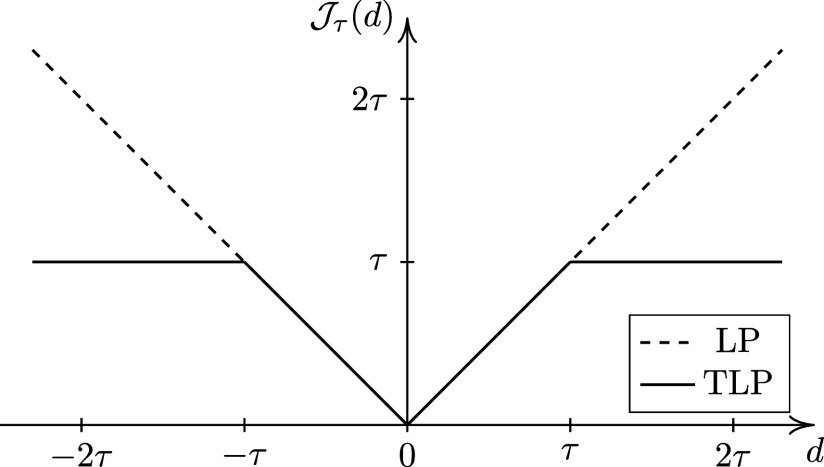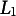 truncated *L*_1_ penalties.

Figure 1 shows both LP and TLP. Note that TLP becomes LP as 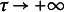 . When 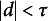 , the two penalties are the same, so both shrink small values to zero. When *d* is already large (i.e., 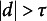 ), TLP is capped at 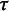 , i.e., it applies a constant penalty when *d* is too large to be shrunk to zero. As a result, TLP has less bias than LP and is hence preferable. Moreover, when 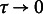 , 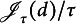 , a rescaled version of the TLP, becomes the ideal 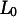 penalty, thus it performs the model selection task of the 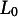 function by providing a computationally efficient surrogate (see Section 2.2 for computational details).

#### 2.1.3 Group pairwise comparison and penalty

Existing regularization methods penalize differences between each focal group and the reference only, while differences among focal groups are disregarded. Instead, our proposed penalty term is 
(3)

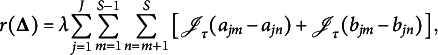

which we call group pairwise TLP because we penalize the item parameter differences between every pair of groups. Similar ideas have been adopted by previous studies, including the fused lasso (Tibshirani et al., 2005) and a grouping pursuit algorithm (Shen & Huang, 2010). In this study, we extend them to accommodate TLP. It is worth noting that (3) imposes a common tuning parameter 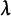 for both the *a* and *b* parameters, a choice also made in prior studies (Belzak & Bauer, 2020; Lyu et al., 2025; Wang et al., 2023). While this approach simplifies the model and computation, it may not yield optimal performance in practice because *a* and *b* have different scales. Using separate tuning parameters (i.e., 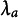 and 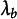 ) could potentially improve performance. However, in this study, we adopt a shared 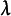 for two reasons. First, it offers greater computational efficiency. Equation (3) already involves two tuning parameters, 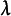 and 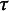 , requiring a two-dimensional grid search. Introducing a third parameter would increase the search space to three dimensions, making computation substantially more intensive. Second, because the 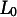 penalty is scale-invariant and TLP approximates the 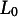 penalty, TLP is less sensitive to variable scales than LP. Indeed, Shen et al. (2012) showed that under certain conditions, TLP achieves consistent variable selection using a common tuning parameter, suggesting that using a common 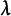 in (3) remains effective in large samples.

Multi-group IRT models with both impact and DIF are not identified, making DIF detection highly dependent on identifying DIF-free items that serve as anchor items. By applying a pairwise penalty across groups, the proposed method imposes stricter penalties on DIF parameters for DIF-free items because it involves comparisons across 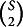 pairs, rather than the 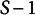 pairwise comparisons between focal groups and a single reference group in traditional approaches. When item parameters among focal groups differ only by a small amount in opposite directions relative to the reference group, existing methods struggle to detect this subtle DIF, even though DIF among focal groups is more substantial. In contrast, the pairwise penalty approach identifies and leverages these larger DIF parameters among focal groups, resulting in more accurate detection. Consider a hypothetical case where there are four groups and Item 1 is DIF-free. For simplicity, we use the LP and focus on the estimates of Item 1’s difficulty parameters, 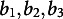 , and 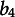 . Traditional regularization methods penalize 

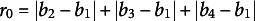

if Group 1 is chosen to be the reference, while our proposed method penalizes 

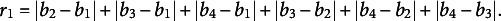

Suppose that the estimates of 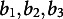 , and 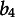 by the traditional method are 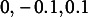 , and 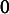 , respectively. Since the penalty term 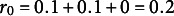 is small, the traditional method fails to shrink both 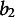 and 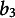 to 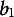 . In contrast, the group pairwise penalty is 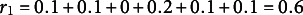 , where focal groups are also directly compared. This larger penalty is more likely to finally result in perfect shrinkage, 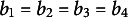 . That is, Item 1 is more likely to be correctly identified as DIF-free and hence work as an anchor under the proposed penalty. As the number of groups increases, the group pairwise penalty will penalize item parameter differences in DIF-free items even more strongly, so our proposed method is expected to have higher accuracy of DIF detection.

In addition, compared to existing DIF detection methods, this novel pairwise penalty is essential for the method to work with *small sample sizes*. That is, the specific type of penalization encourages similarity across groups, hence, a group with a small sample size (e.g., a certain unique intersectional identity) can leverage data from other larger groups it shares common identities with. The idea also bears resemblance to fair regression in machine learning (Berk et al., 2017).

#### 2.1.4 Optimization problem for model estimation

Summarizing the discussions in Sections 2.1.1–2.1.3, our goal is to maximize the penalized log marginal likelihood function 
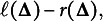
 or equivalently to minimize 

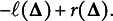

Imposing penalties on differences between item parameters rather than parameters themselves makes it challenging to directly solve the optimization problem, so we introduce the difference parameters 

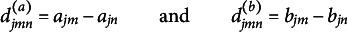

as the item parameter differences to be penalized, and define 

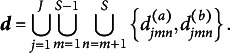

Then, under the reparametrization, the penalty term becomes 

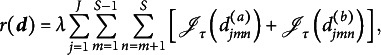

and hence, we can estimate the same model by solving the constrained optimization problem 
(4)

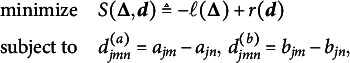

where 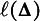 is defined in (2). The optimization problem (4) presents two challenges: (a) the TLP term 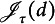 is non-differentiable and non-convex and (b) the constraints defining 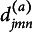 and 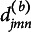 . We address these computational issues in the next section.

### 2.2 Computational algorithm

Although the TLP term 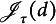 is not a convex function, it is piecewise linear and can be decomposed into a difference of two convex functions as 

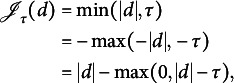

which allows us to use DC programming to gain computational advantage (Shen et al., 2012; Xu & Shang, 2018). In particular, following Ma et al. (2023), we consider the following DC decomposition: 

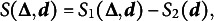

where 

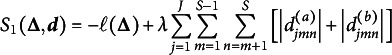

and 

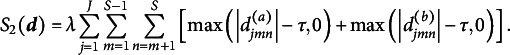



During the estimation, we iteratively construct a sequence of upper approximations of 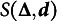 by replacing 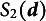 at iteration 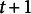 with its minorization, 

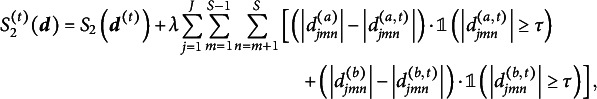

which reduces the objective function to 

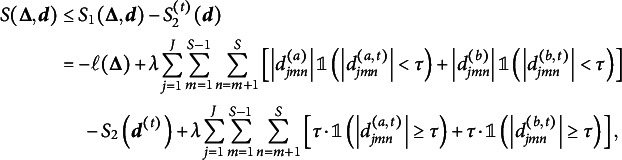

whose last two terms can be omitted because they do not involve any parameters in 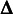 or 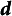 . Letting 

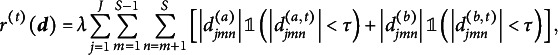

Our objective function to be minimized at iteration 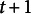 becomes 

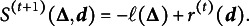



To deal with the constraints in (4), we apply ADMM (Boyd et al., 2010), which leads to the augmented Lagrangian 

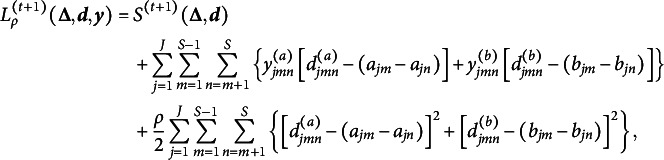

where 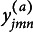 and 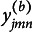 are dual variables (or Lagrange multipliers) of their corresponding constraints and 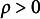 is a penalty parameter. Letting 
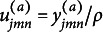
 and 
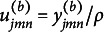
 be the scaled dual variables, ADMM can be expressed as (Boyd et al., 2010, p. 15) 
(5)

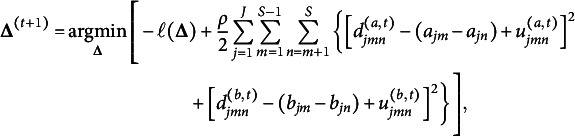



(6)

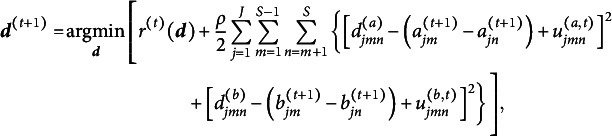



(7)

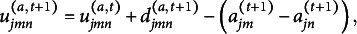



(8)

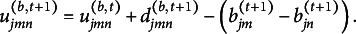

Although (6) has closed-form solutions 

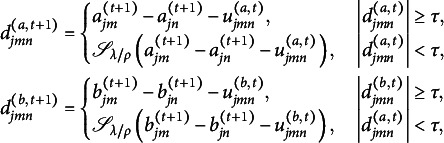

where 

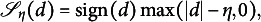

there is no closed-form solution for (5). Since (5) involves integration with respect to latent variables, we use Gaussian quadrature to approximate the integrals and apply the EM algorithm for estimation. In the E-step, we compute the posterior distribution of the latent variable 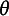 for each respondent. In the M-step, we minimize the expectation of (5) with respect to 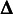 and update other parameters using (6), (7), and (8). There are closed-form update rules for impact parameters 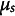 and 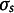 , and the L-BFGS algorithm (Liu & Nocedal, 1989) is applied for updating item parameters 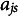 and 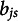 . We fix 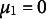 and 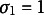 for model identification. Our final algorithm is shown in Algorithm 1, where the convergence criterion can be defined such that the absolute change in every parameter between the current and the last iterations is smaller than some small value, such as 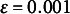 .

**Figure:**
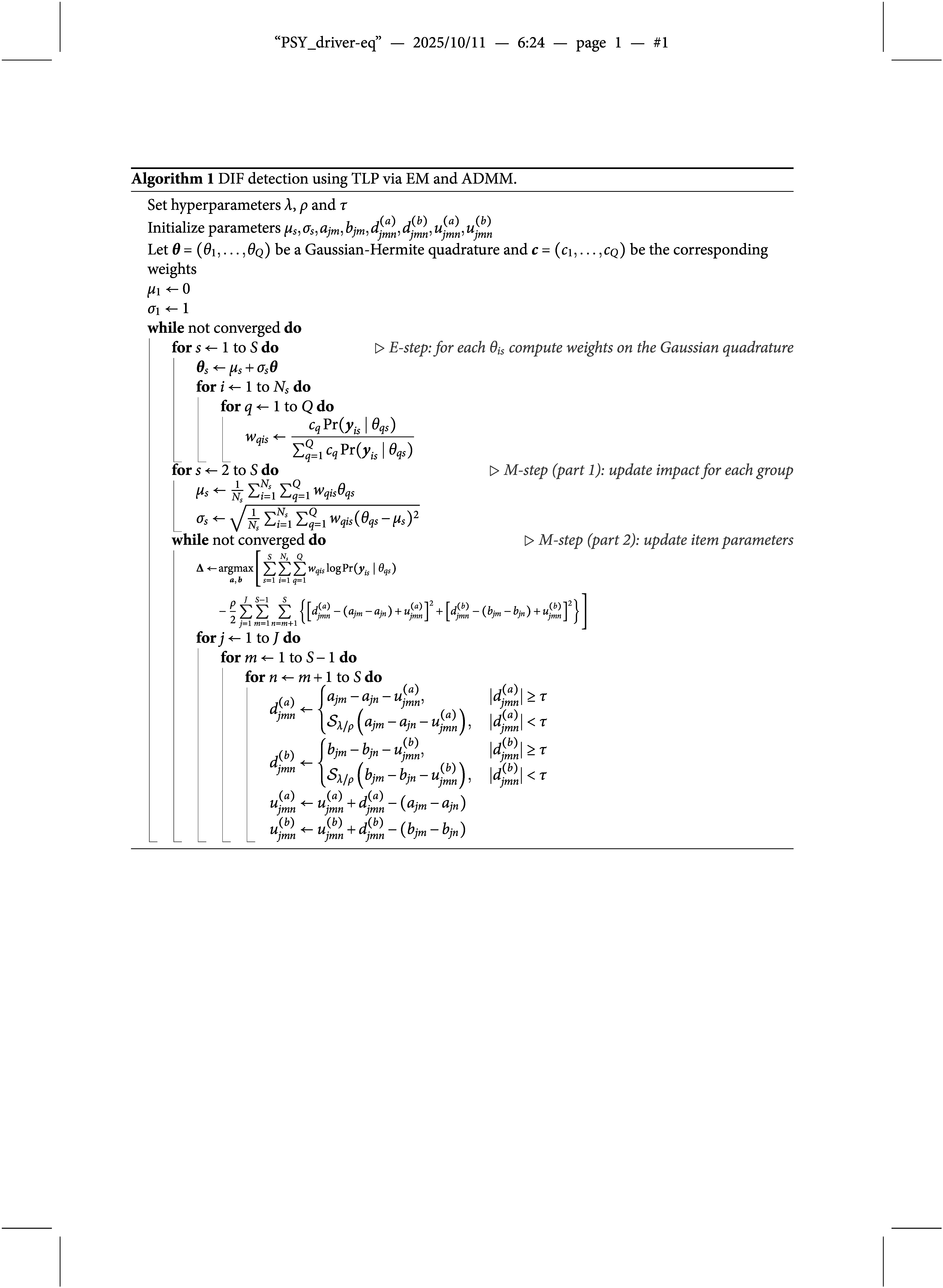

To initialize the algorithm, we first run Algorithm 1 with 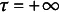 to obtain initial values, where TLP becomes LP in this case and the corresponding optimization problem is easier to solve due to the convexity of LP.

**Figure 2:**
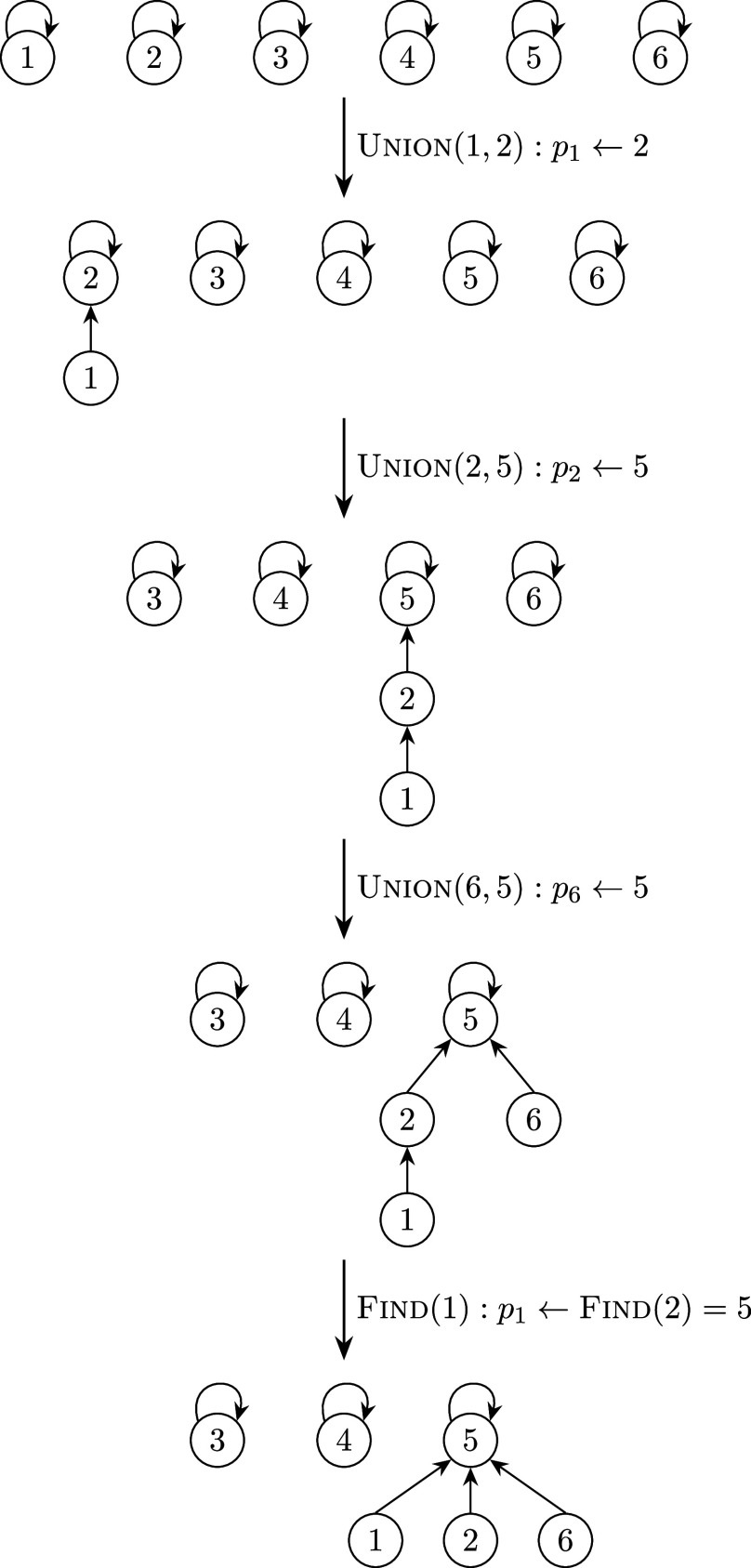
An illustration of the union-find data structure.

When the algorithm converges, no DIF is detected between groups *m* and *n* on item *j* if and only if 
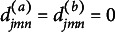
 . If 
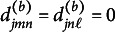
 for 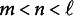 , then analytically 
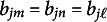
 because of the constraints in (4), and hence 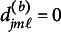 . Numerically, however, it is possible that both 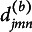 and 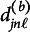 have already been shrunk to zero while 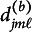 still takes a small nonzero value because the algorithm does not explicitly check the equality transitivity on the one hand, and on the other hand, the algorithm stops when the convergence criterion is met, which only leads to an approximation of the true extreme point. To reduce numerical error and guarantee the transitive property, we directly assign 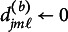 in such cases. For each item parameter, we initially let each group form a cluster, and then each pair of clusters with the same item parameter is collapsed into a bigger cluster. Finally, each cluster consists of groups that share the same item parameter. This is implemented using the union-find data structure (Kleinberg & Tardos, 2005), which is widely used in computer science literature. Figure 2 shows an example of how the union-find data structure works. Each cluster is a tree whose root is its representative group *x*, which satisfies that 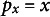 . Here, 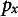 indicates the parent of *x*. In the beginning, each group forms a single cluster. To collapse clusters, including Groups 1 and 2, we let 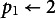 , so they form a bigger cluster whose representative is Group 2. To collapse clusters, including Groups 2 and 5, we let 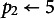 , so Group 1 indirectly points to the new representative, Group 5, by going through Group 2. Therefore, the representative of the cluster, including Group 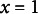 , can be obtained by going along the path indicated by *p*, i.e., 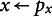 repeatedly until 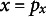 . After we reach Group 5 from Group 1, we let 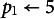 because there is no need to go through Group 2 again the next time we start from Group 1. That is, every group along the path can point directly to the representative in order to save time for future operations, a technique called path compression. The procedure for collapsing groups to guarantee the transitive property is shown in Algorithm 2 and we apply it to both 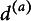 and 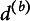 after Algorithm 1 converges.

**Figure:**
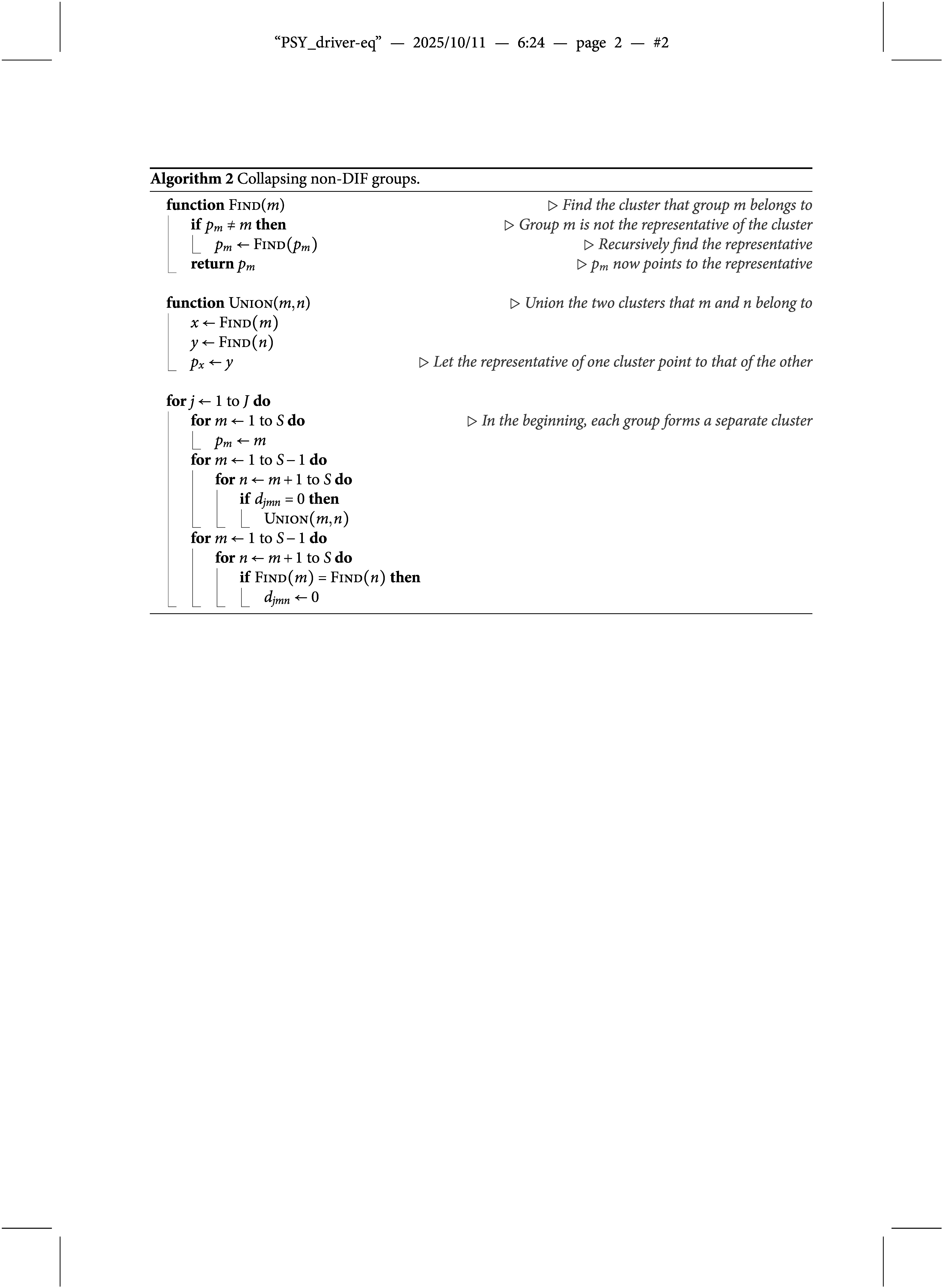

To select the model with the best tuning parameters 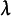 and 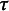 , we try different values and then choose the one with the lowest Bayesian information criterion (BIC), 

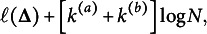

where 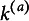 and 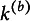 are the numbers of distinct 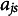 and 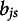 parameters. Note that the value 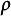 mainly affects the convergence rate but has little effect on the accuracy (Boyd et al., 2010).

## 3 Simulation study

We consider two cases with 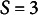 and 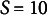 , respectively. In both cases, there are 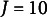 items and the item parameters for group 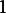 follow 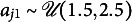 and 
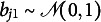
 . Moreover, the first 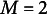 or 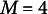 items have DIF. The simulation settings are shown in Tables 1 and 2. Besides DIF, the impact is also simulated, although we assume equal variance and only vary means across groups.

**Table 1:** Impact and DIF parameters for 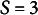 groups

s	1	2	3
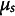	0	1	−1
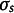	1	1	1
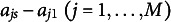	0	1	−1
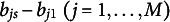	0	1.5	−1.5

**Table 2:** Impact and DIF parameters for 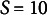 groups

s	1	2	3	4	5	6	7	8	9	10
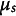	0	0	1	1	1	1	−1	−1	−1	−1
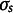	1	1	1	1	1	1	1	1	1	1
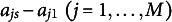	0	0	0.5	0.5	−0.5	−0.5	1	1	−1	−1
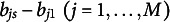	0	0	1	−1	1	−1	1.5	−1.5	1.5	−1.5

We run 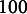 replications for each setting. For each replication, we fix 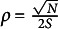 , fit the model with different combinations of 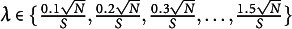 and 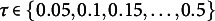 , and pick the one that leads to the lowest BIC. The convergence criterion is that the absolute change in each parameter is smaller than 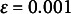 , and initial parameters are obtained by the proposed method with LP (i.e., TLP with 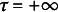 ). The true and false positive rates among the 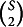 pairs of groups are computed and summarized across replications.

For comparison, we also apply two regularized DIF detection methods from the R packages VEMIRT (Lyu et al., 2025) and regDIF (Belzak, 2023). The VEMIRT package implements the importance-weighted Gaussian variational EMM (IW-GVEMM) algorithm with the LP, which has been shown to achieve accurate DIF detection with efficient computation (Lyu et al., 2025). The regDIF package supports both the LP and the minimax concave penalty (MCP), and we specify MCP for this simulation study. MCP is an alternative to TLP for reducing the estimation bias of the LP by keeping the penalty constant when the parameter value is large (Zhang, 2010). Similar to TLP, which includes a tuning parameter 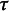 , MCP has a tuning parameter 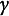 . We retain its default value of 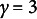 because the algorithm becomes computationally slow even without fine-tuning 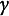 . In a few replications, regDIF failed to fully converge, suggesting that MCP’s performance could be improved with an optimal choice of 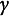 . Both methods require a reference group; therefore, we run them *S* times, each time letting a different group be the reference to allow pairwise comparisons. Then, an item is flagged as DIF between two groups if DIF is detected for both groups when the other one is the reference group.^1^

### 3.1 Simulation I: Balanced design

**Figure 3:**
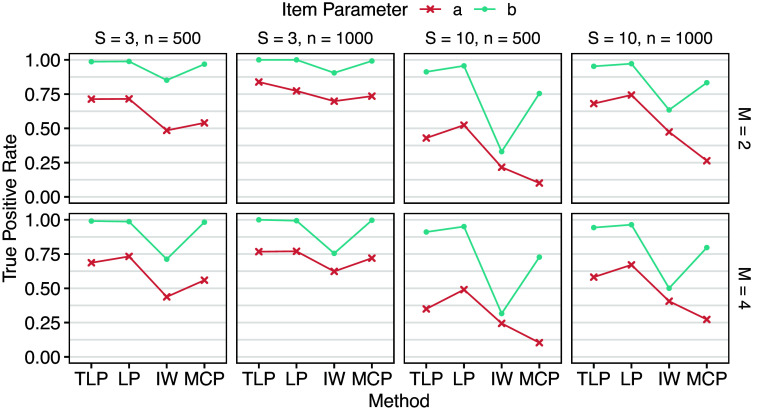
Mean true positive rates across replications of Simulation I.

**Figure 4:**
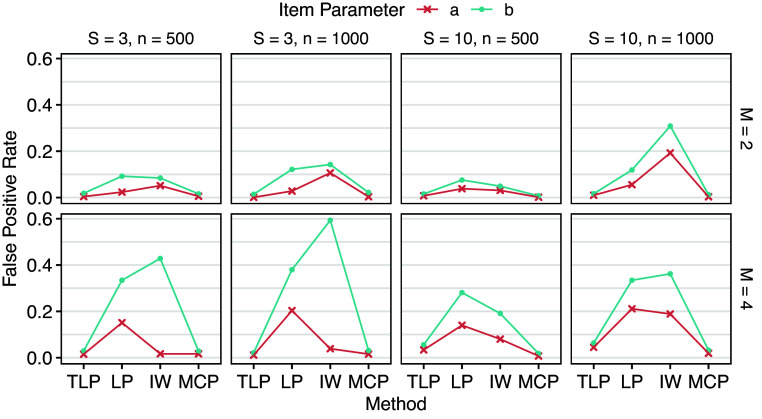
Mean false positive rates across replications of Simulation I.

Under the balanced design, each group has either 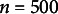 or 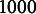 respondents, and the total sample size is 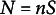 . DIF detection results are shown in Tables 3 and 4, and Figures 3 and 4 provide corresponding visualizations. DIF on *a* (slopes) generally has lower true and false positive rates than DIF on *b* (intercepts), suggesting that all the methods are more sensitive to group differences in *b*. Fixing *S* (number of groups), larger *n* (number of respondents in each group) leads to higher true positive rates, which is expected. However, false positive rates also tend to increase as *n* increases for LP and IW-GVEMM, while TLP and MCP consistently have better performance with larger sample sizes. Fixing *n*, larger *S* leads to lower true positive rates. This is not surprising because we are conducting 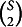 group pairwise comparisons. When the number of DIF items increases from 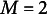 to 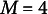 , the performance of all methods becomes worse, particularly due to higher false positive rates. When DIF items constitute a large proportion, such as 40%, model identifiability becomes a greater concern. In such cases, DIF in item parameters may instead be absorbed into impact to maximize the penalized marginal likelihood function. This agrees with Wang et al. (2023), who found that the bias due to lasso gradually accumulates during the EM estimation process, and hence they proposed the EMM algorithm to reduce bias after each EM iteration. Since TLP and MCP closely approximate the 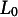 penalty and do not strongly penalize large DIF parameters, such issue is less likely to happen. As a result, TLP and MCP maintain reasonable false positive rates, whereas LP and IW-GVEMM exhibit excessively high false positive rates, making them impractical for reliable DIF detection.

**Table 3:** Means (standard deviations) of true positive rates across replications of Simulation I

			TLP		LP		IW-GVEMM		MCP	
*S*	*M*	*n*	*a*	*b*	*a*	*b*	*a*	*b*	*a*	*b*
		500	0.713 (0.155)	0.987 (0.045)	0.715 (0.099)	0.988 (0.043)	0.485 (0.215)	0.852 (0.153)	0.540 (0.200)	0.968 (0.070)
	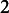	1000	0.838 (0.126)	1.000 (0.000)	0.773 (0.115)	1.000 (0.000)	0.698 (0.178)	0.905 (0.109)	0.735 (0.177)	0.992 (0.044)
			500	0.687 (0.146)	0.991 (0.026)	0.733 (0.117)	0.987 (0.033)	0.438 (0.169)	0.712 (0.102)	0.559 (0.189)	0.983 (0.038)
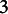	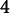	1000	0.767 (0.102)	1.000 (0.000)	0.770 (0.082)	0.993 (0.035)	0.623 (0.149)	0.755 (0.083)	0.720 (0.145)	0.997 (0.016)
		500	0.430 (0.220)	0.912 (0.042)	0.524 (0.189)	0.956 (0.020)	0.216 (0.059)	0.330 (0.090)	0.101 (0.073)	0.754 (0.069)
	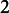	1000	0.681 (0.159)	0.953 (0.036)	0.743 (0.153)	0.972 (0.017)	0.474 (0.100)	0.635 (0.072)	0.264 (0.126)	0.833 (0.048)
		500	0.350 (0.197)	0.911 (0.044)	0.490 (0.153)	0.951 (0.032)	0.244 (0.039)	0.316 (0.059)	0.103 (0.063)	0.727 (0.109)
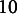	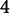	1000	0.582 (0.189)	0.943 (0.041)	0.671 (0.162)	0.964 (0.017)	0.406 (0.063)	0.501 (0.048)	0.273 (0.136)	0.797 (0.127)

**Table 4:** Means (standard deviations) of false positive rates across replications of Simulation I

			TLP		LP		IW-GVEMM		MCP	
*S*	*M*	*n*	*a*	*b*	*a*	*b*	*a*	*b*	*a*	*b*
		500	0.004 (0.022)	0.018 (0.035)	0.023 (0.049)	0.092 (0.090)	0.051 (0.053)	0.084 (0.072)	0.005 (0.020)	0.016 (0.040)
	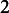	1000	0.001 (0.008)	0.014 (0.036)	0.028 (0.051)	0.122 (0.102)	0.106 (0.057)	0.142 (0.085)	0.003 (0.018)	0.022 (0.039)
		500	0.015 (0.042)	0.029 (0.068)	0.151 (0.133)	0.334 (0.176)	0.017 (0.030)	0.428 (0.126)	0.017 (0.033)	0.028 (0.050)
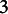	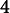	1000	0.011 (0.034)	0.021 (0.049)	0.028 (0.051)	0.203 (0.153)	0.380 (0.183)	0.593 (0.127)	0.016 (0.040)	0.031 (0.048)
		500	0.008 (0.005)	0.015 (0.010)	0.038 (0.040)	0.076 (0.059)	0.031 (0.025)	0.049 (0.040)	0.001 (0.002)	0.007 (0.006)
	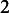	1000	0.010 (0.009)	0.016 (0.009)	0.055 (0.042)	0.119 (0.080)	0.193 (0.036)	0.309 (0.060)	0.003 (0.003)	0.013 (0.010)
		500	0.034 (0.033)	0.056 (0.041)	0.140 (0.085)	0.281 (0.119)	0.080 (0.023)	0.191 (0.039)	0.007 (0.009)	0.018 (0.012)
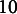	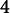	1000	0.045 (0.042)	0.064 (0.044)	0.211 (0.100)	0.334 (0.120)	0.189 (0.034)	0.362 (0.033)	0.019 (0.016)	0.032 (0.020)

LP has higher true positive rates than TLP in most cases, especially when there are more groups, at the cost of much higher and almost unacceptable false positive rates. Since the LP shrinks all the DIF parameters toward zero in a way that larger parameters are penalized more, DIF parameter estimates are known to be biased (Wang et al., 2023). As a result, BIC, which is based on maximum likelihood, has difficulty finding the best model under LP. In contrast, TLP becomes constant for large DIF parameters, so they are not strongly biased toward zero. That is, parameter estimates by TLP are more accurate and less biased. IW-GVEMM performs worse than both TLP and LP: it has the lowest true positive rates and high false positive rates, suggesting that IW-GVEMM is not suitable for group pairwise DIF detection. As discussed earlier, this difference is mainly due to the differences in the ability to identify DIF-free items that work as anchors for DIF detection. TLP and LP impose a stronger penalty on item parameter differences in DIF-free items compared to IW-GVEMM, so they identify DIF-free items more accurately. In addition, we notice that IW-GVEMM sometimes fails to find anchor items when impact is large, but this model identifiability issue becomes less of a problem for IW-GVEMM when there is less impact among the groups (Lyu et al., 2025). Among all methods, MCP yields the lowest false positive rates. However, this comes at the cost of being conservative, as reflected in its lower true positive rates compared to TLP and LP. In particular, MCP has difficulty in detecting DIF in slopes. Since both TLP and MCP approximate the 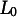 penalty, these results suggest the importance of imposing a group pairwise penalty, rather than shrinking all focal groups toward a prespecified reference. In summary, TLP demonstrates superior overall performance over LP, IW-GVEMM, and MCP.

### 3.2 Simulation II: Unbalanced design

**Figure 5:**
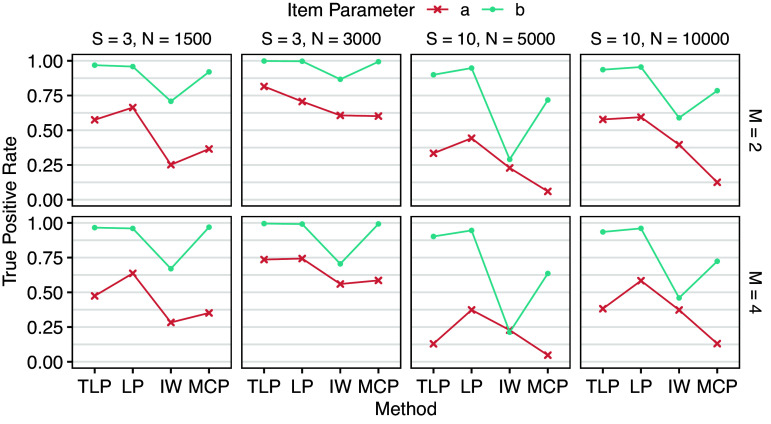
Mean true positive rates across replications of Simulation II.

**Figure 6:**
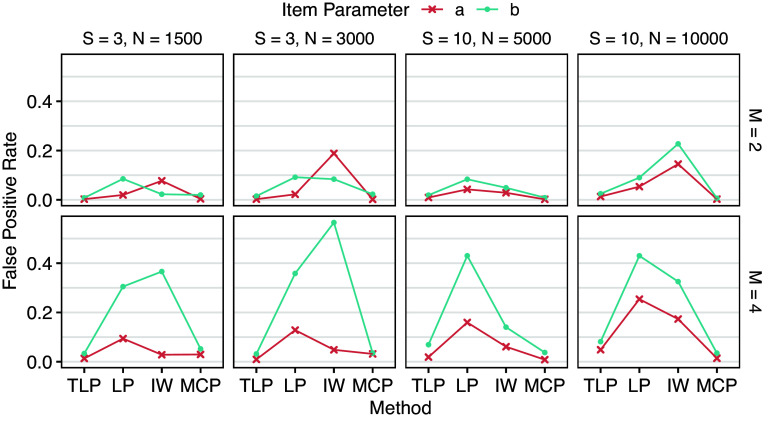
Mean false positive rates across replications of Simulation II.

The simulation setting of the unbalanced design is the same as the balanced design except that groups have different sizes. Tables 5 and 6 show the proportion of group sizes relative to *N*. DIF detection results are shown in Tables 7 and 8, and Figures 5 and 6 provide corresponding visualizations. Basically, they show the same patterns as Tables 3 and 4, but the unbalanced design results in lower true positive rates than the balanced design for all the methods. The false positive rates of TLP, LP, and MCP tend to become higher; those of IW-GVEMM are lower, although still too high to be useful. The reason is that some groups are so small that their item parameters become very difficult to estimate and tend to be shrunk toward other groups. Still, TLP turns out to work well, especially on detecting DIF on intercepts.

**Table 5:** Group sizes for 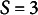 groups under unbalanced design

*s*	1	2	3
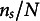	0.6	0.2	0.2

**Table 6:** Group sizes for 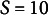 groups under unbalanced design

*s*	1	2	3	4	5	6	7	8	9	10
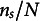	0.1	0.1	0.15	0.15	0.05	0.05	0.15	0.15	0.05	0.05

**Table 7:** Means (standard deviations) of true positive rates across replications of Simulation II

			TLP		LP		IW-GVEMM		MCP	
*S*	*M*	*N*	*a*	*b*	*a*	*b*	*a*	*b*	*a*	*b*
		1500	0.575 (0.261)	0.968 (0.070)	0.663 (0.152)	0.958 (0.090)	0.252 (0.246)	0.708 (0.194)	0.365 (0.263)	0.920 (0.115)
	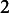	3000	0.815 (0.148)	0.998 (0.017)	0.707 (0.092)	0.997 (0.023)	0.607 (0.266)	0.867 (0.128)	0.602 (0.271)	0.993 (0.033)
		1500	0.474 (0.279)	0.966 (0.067)	0.637 (0.194)	0.960 (0.060)	0.283 (0.165)	0.670 (0.117)	0.352 (0.219)	0.969 (0.061)
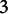	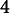	3000	0.736 (0.174)	0.995 (0.023)	0.743 (0.091)	0.992 (0.028)	0.560 (0.160)	0.705 (0.106)	0.586 (0.222)	0.993 (0.038)
		5000	0.334 (0.266)	0.899 (0.043)	0.442 (0.199)	0.947 (0.024)	0.228 (0.044)	0.290 (0.081)	0.060 (0.065)	0.717 (0.088)
	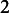	10000	0.578 (0.259)	0.936 (0.036)	0.594 (0.216)	0.955 (0.024)	0.396 (0.078)	0.589 (0.080)	0.125 (0.093)	0.785 (0.076)
		5000	0.129 (0.184)	0.902 (0.033)	0.373 (0.179)	0.946 (0.026)	0.228 (0.040)	0.213 (0.065)	0.048 (0.042)	0.636 (0.162)
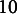	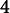	10000	0.382 (0.256)	0.935 (0.032)	0.584 (0.156)	0.960 (0.016)	0.373 (0.044)	0.460 (0.064)	0.131 (0.097)	0.724 (0.165)

**Table 8:** Means (standard deviations) of false positive rates across replications of Simulation II

			TLP		LP		IW-GVEMM		MCP	
*S*	*M*	*N*	*a*	*b*	*a*	*b*	*a*	*b*	*a*	*b*
		1500	0.003 (0.014)	0.008 (0.028)	0.020 (0.045)	0.085 (0.094)	0.077 (0.067)	0.023 (0.033)	0.004 (0.012)	0.020 (0.039)
	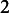	3000	0.003 (0.014)	0.015 (0.032)	0.022 (0.040)	0.092 (0.094)	0.188 (0.069)	0.084 (0.066)	0.002 (0.008)	0.023 (0.039)
		1500	0.013 (0.036)	0.033 (0.062)	0.094 (0.112)	0.305 (0.163)	0.028 (0.039)	0.366 (0.142)	0.029 (0.046)	0.052 (0.061)
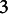	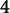	3000	0.009 (0.033)	0.032 (0.075)	0.128 (0.153)	0.358 (0.185)	0.048 (0.057)	0.565 (0.161)	0.032 (0.060)	0.035 (0.052)
		5000	0.009 (0.011)	0.019 (0.018)	0.042 (0.036)	0.083 (0.062)	0.029 (0.017)	0.049 (0.028)	0.002 (0.003)	0.009 (0.008)
	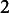	10000	0.013 (0.013)	0.025 (0.027)	0.053 (0.042)	0.090 (0.065)	0.145 (0.029)	0.227 (0.047)	0.003 (0.003)	0.006 (0.007)
		5000	0.019 (0.030)	0.069 (0.054)	0.159 (0.090)	0.430 (0.155)	0.061 (0.023)	0.140 (0.035)	0.008 (0.009)	0.037 (0.037)
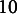	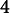	10000	0.049 (0.053)	0.082 (0.064)	0.254 (0.103)	0.430 (0.140)	0.173 (0.024)	0.325 (0.039)	0.014 (0.016)	0.035 (0.027)

## 4 Applications

In this section, we apply our proposed methods to two real-world datasets, one from a large-scale international assessment, and the other one from an adaptive language assessment.

### 4.1 Cross-economy data from PISA

The Programme for International Student Assessment (PISA) is an international large-scale assessment for 15-year-old students created by OECD. A subset of PISA 2018 science data is analyzed, which includes 6,319 students from ten countries or economies and 19 dichotomous items. We consider countries and economies as groups for DIF detection because, in an international assessment like PISA, it is crucial to ensure that test items function consistently and equitably for students across all countries and economies. Failing to do so would render any international comparison based on the assessment results invalid. Table 9 shows these ten countries or economies. For simplicity, we will refer to both countries and economies as “economies” throughout this discussion.

**Table 9:** Countries and economies in the PISA analysis

	Abbreviation	Country or Economy
1	EST	Estonia
2	FIN	Finland
3	FRA	France
4	GBR	United Kingdom
5	GEO	Georgia
6	GRC	Greece
7	HKG	Hong Kong
8	HRV	Croatia
9	HUN	Hungary
10	IDN	Indonesia

**Figure 7:**
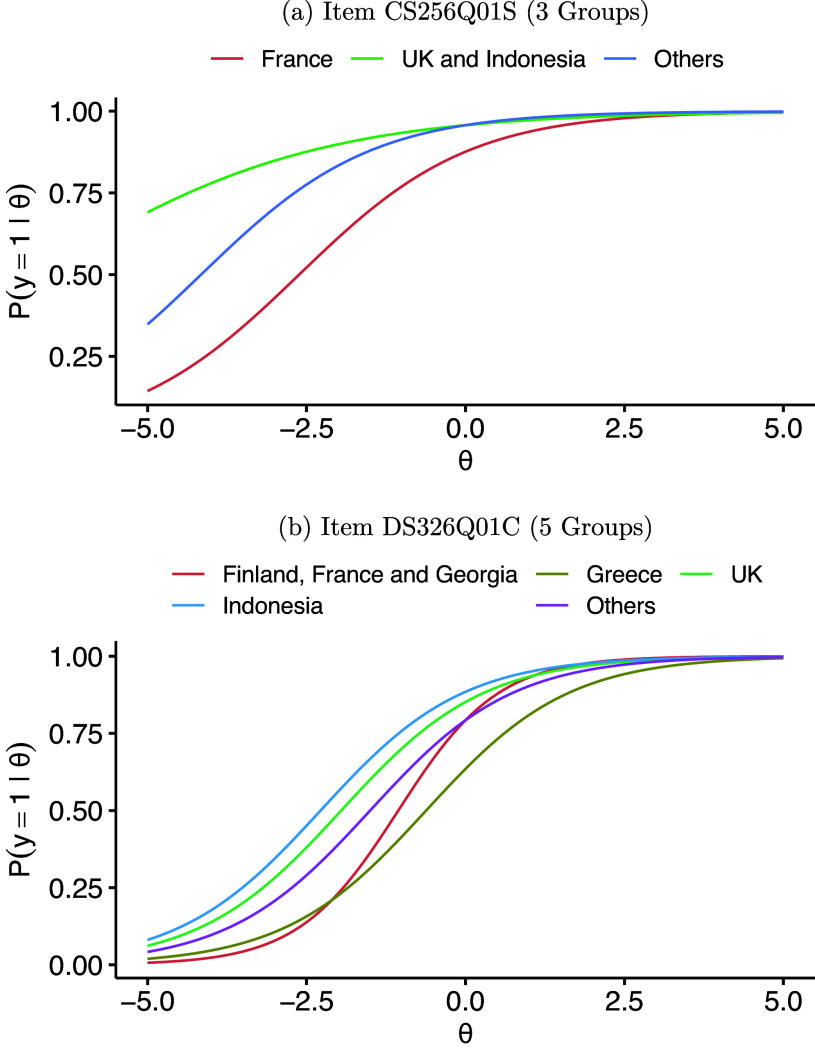
ICCs of PISA items.

TLP suggests that all 19 items have some level of DIF, i.e., all these items will have at least two sets of different parameters. For each item, we collapse economies that do not have DIF among them and compute the number of distinct groups, which are shown in Table 10. Most items divide the ten economies into 3–6 homogeneous groups. Figure 7 shows the item characteristic curves (ICCs) of two items that divide the ten economies into 3 and 5 groups, respectively. It is clear that the ICCs are quite distinct across groups.

**Table 10:** Frequency table of numbers of distinct groups

Number of distinct groups	2	3	4	5	6	7	8
Number of items	1	5	2	6	3	1	1

**Figure 8:**
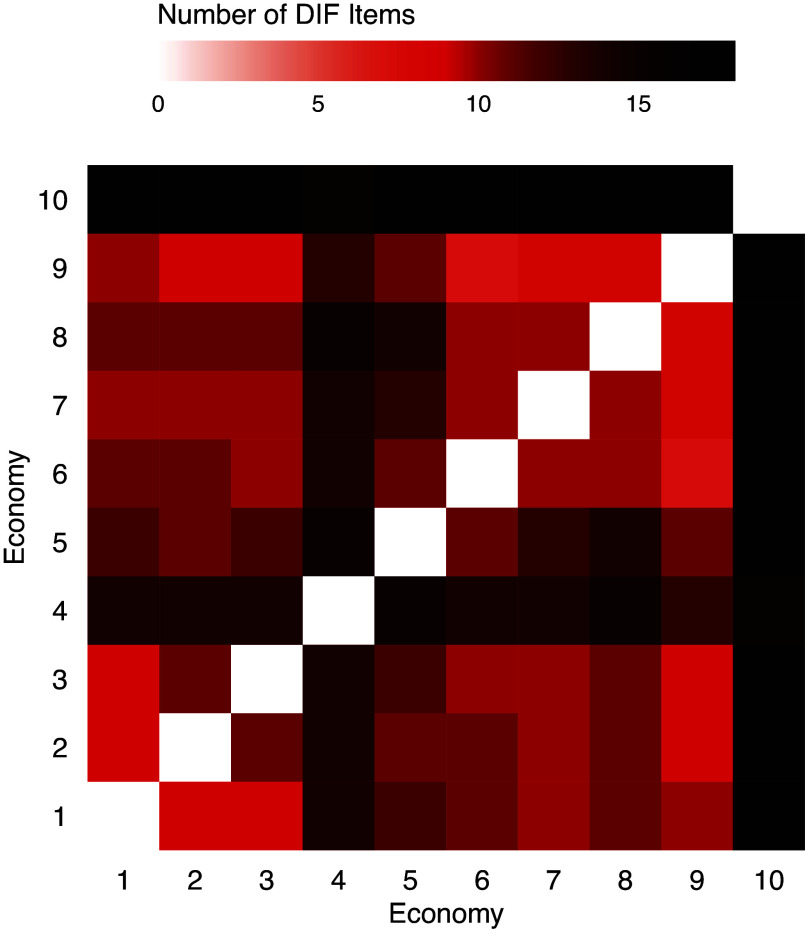
Numbers of DIF items between pairs of economies using TLP with 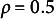 .

The sample size and estimated impact of each economy are shown in Table 11, and pairwise comparison DIF results are shown in Figure 8. Most economy pairs have DIF in more than ten items, which account for more than half of the total items. Economies 10 (Indonesia) and 4 (United Kingdom) tend to have the most DIF items. As these are the two largest groups in the data, their item parameter estimates tend to be more accurate and easier to separate from other groups. It is also worth noting that the latent trait distributions differ a lot across economies. In particular, the mean math ability of respondents from Economy 10 (Indonesia) is much lower than other economies.

**Table 11:** Sample sizes and estimated impact of economies

*s*	1	2	3	4	5	6	7	8	9	10
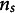	586	607	505	1347	237	404	512	401	471	1249
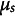	0	−0.20	−0.30	−0.36	−1.35	−1.01	−0.08	−0.63	−0.64	−1.63
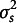	1	1.40	0.77	1.37	0.79	0.78	0.86	1.12	1.13	0.73

### 4.2 An adaptive language assessment

To demonstrate the flexibility of the proposed method, we also consider a data set from a large-scale adaptive language assessment. Unlike the PISA data, these assessment data have a unique feature: a large item bank relative to sample size per item because the items were generated with the assistance of AI and the assessment is adaptive. As a result, the response matrix of respondent by item is very sparse, and the overall sample size is large. Since this is a proprietary, high-stakes assessment, the data were provided to us from the test owner, and they pulled the data in such a way that each item was answered by at least 500 respondents. This ensures a sufficient sample size per item, especially when we evaluate the DIF on multiple subgroups. However, this data extraction scheme results in incomplete responses per respondent. Hence, latent ability estimates from a complete operational test, derived using a proprietary psychometric model, are provided alongside the response data. Based on these estimates, four respondents whose ability estimates were more than five standard deviations below the mean were excluded. However, these ability estimates are not used in the subsequent DIF analysis. Instead, we apply our proposed method to the response data without relying on the original ability estimates, as they may have been contaminated by the presence of DIF.

One specific item type was explored. For this type, respondents are asked to type the missing letters to complete the text. That is, they will fill in the blanks of unfinished words in a passage. This item type aims to measure reading, literacy, and comprehension. We analyze subtasks that are scored as 0 or 1. Groups are formed by the interaction of self-reported native language and gender. We drop people from the non-binary gender category because they only account for less than 0.1% of the respondents in the data.

**Figure 9:**
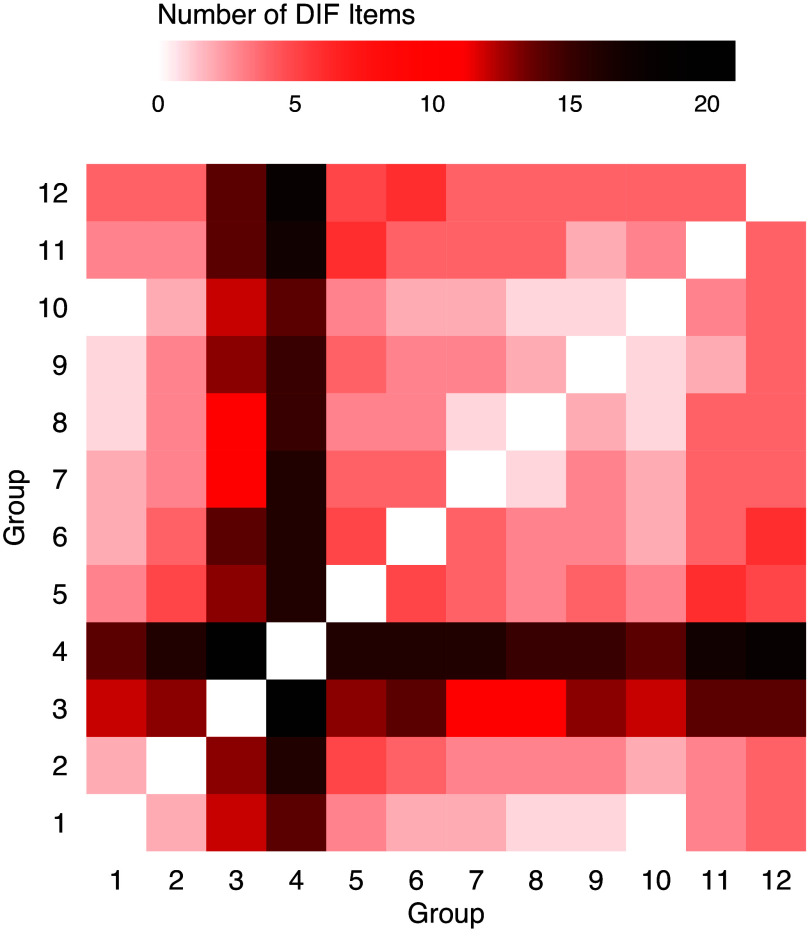
Numbers of DIF items between pairs of groups in the language assessment using TLP with 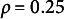 .

The six largest native language groups (Chinese–Mandarin, English, Spanish, Arabic, Hindi, and Portuguese) form 12 groups by interacting with gender. Table 12 shows the basic information for each group. This subsample has 3,734 respondents and 234 items, each item is answered by at least 500 respondents. DIF is detected in 27 of 234 items and pairwise comparison results are shown in Figure 9. The two Chinese (Mandarin) groups have the most DIF items compared to other linguistic groups, and somewhat surprisingly, there are 21 DIF items between the Chinese female and Chinese male groups. Besides, when each of the eight non-Chinese and non-Spanish groups is compared with the Spanish groups, approximately four DIF items are consistently identified, whereas comparisons among these eight groups themselves yield fewer DIF items overall.

**Table 12:** Groups in the language assessment

*s*	Native language	Gender	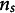	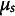	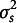
1	Arabic	Female	212	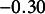	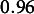
2	Arabic	Male	223	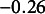	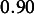
3	Chinese – Mandarin	Female	502	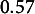	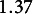
4	Chinese – Mandarin	Male	527	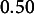	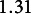
5	English	Female	409	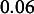	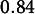
6	English	Male	395	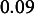	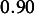
7	Hindi	Female	152	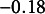	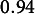
8	Hindi	Male	238	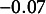	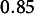
9	Portuguese	Female	182	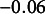	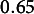
10	Portuguese	Male	161	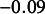	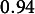
11	Spanish	Female	370	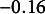	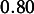
12	Spanish	Male	363	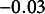	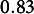

*Note*: Group means ( 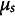 ) and variances ( 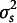 ) are computed from the original ability estimates, which are provided for reference only and not used in the DIF analysis.

There are potentially two caveats when interpreting the results. First, because the response data matrix is sparse, meaning the number of items answered by each respondent varies and is sometimes very small, directly estimating latent abilities from the data may lead to inaccurate results. Second, we treat each subtask as independent, ignoring the innate nested structure (i.e., subtasks are nested within a paragraph). Hence, we use this data set to demonstrate that our algorithm can work on large sparse data sets, but the conclusions drawn therefrom should be further validated based on item content.

**Figure 10:**
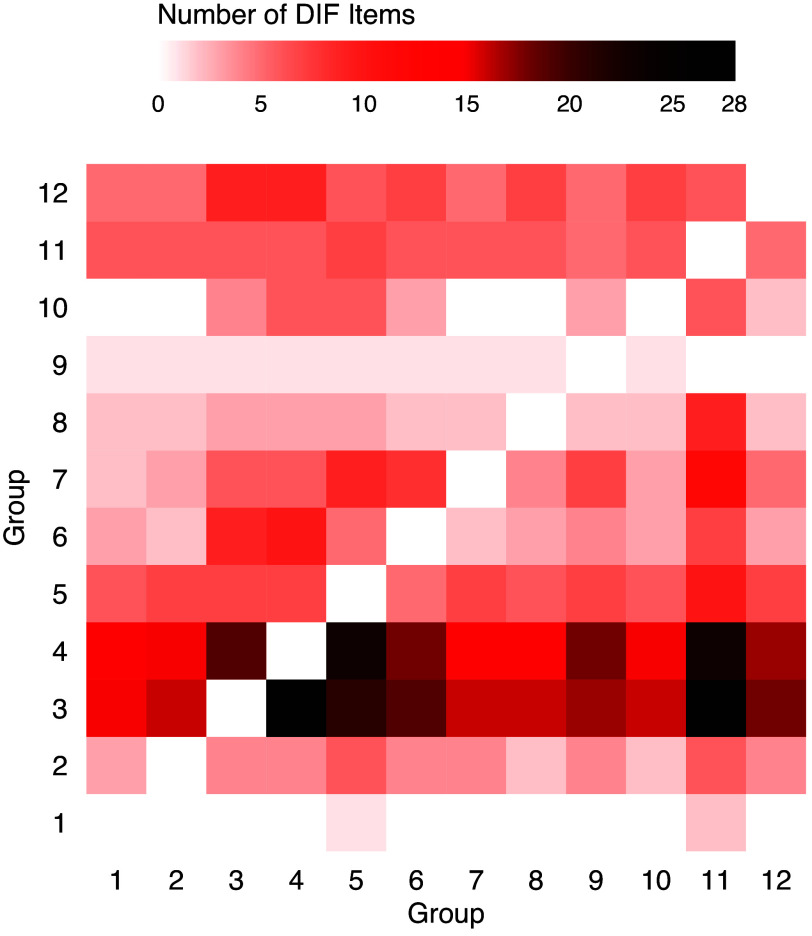
Numbers of DIF items between pairs of groups in the language assessment using IW-GVEMM.

To further verify the findings in Figure 9, we apply IW-GVEMM from the VEMIRT package to the same data set for DIF detection. The IW-GVEMM algorithm is chosen as a reference because the variational method avoids the reliance on quadrature-based integral (or Monte Carlo integral), and since it only contains one tuning parameter, the IW-GVEMM method is computationally much faster than many other methods. We are unable to apply the regDIF package because, as of version 1.1.1, it does not work with item responses containing missing values. The results are shown in Figure 10. Unlike the simulation study, the matrix here is not symmetrized for a more detailed view of the output from IW-GVEMM. Each column indicates the numbers of DIF items when the group corresponding to this column is the reference. While Figure 10 reveals certain similarities with Figure 9, noticeable differences in the overall pattern are also evident. The most notable similarity is that both analyses indicate a high number of DIF items between Groups 3 and 4 and the remaining groups. However, IW-GVEMM only detects this pattern when groups other than Groups 3 and 4 serve as the reference. This observation again emphasizes the key issue with the traditional approach, which relies on a reference group and overlooks other group pairs: when either Group 3 or 4 is used as the reference, it tends to overly shrink all other groups toward itself, leading to an underestimation of DIF effects. In general, there are fewer DIF items detected in Figure 10 compared to Figure 9, which is consistent with the simulation finding that the new method is more powerful in detecting DIF, especially when there are multiple small groups.

## 5 Discussion

In this study, we propose a novel regularization approach for detecting DIF in 2PL models. The method employs a TLP applied to the differences in item parameters across all group pairs, thereby addressing several limitations of existing techniques. Standard 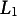 penalties are known to overly shrink large DIF parameters toward zero, leading to biased estimates. In contrast, the TLP is designed to remain constant for large differences, effectively mitigating this bias and allowing for more accurate estimation of substantial DIF effects. A key innovation of our approach lies in its treatment of group comparisons. Traditional methods typically require the specification of a reference group and shrink all focal groups toward it. This practice introduces asymmetry and may lead to unfair DIF detection, as it privileges the reference group and prevents direct comparisons among focal groups. Our method avoids this issue by applying a group pairwise penalty structure, enabling symmetric, interpretable, and direct comparisons between all groups. These advantages make the proposed method particularly effective for detecting DIF in settings involving a large number of small groups. Through simulations, we demonstrate that the proposed method consistently outperforms existing approaches, particularly due to its ability to correctly identify anchor items. This advantage arises from the design of the penalty.

Ensuring that test items are free from DIF is essential for maintaining fairness and validity in educational and psychological assessments. As the development and integration of information technology continue to transform the field of assessment, increasingly large and diverse item response datasets are becoming available. These data often come from large-scale testing programs involving wide-ranging populations, and include items of growing complexity, some even generated by artificial intelligence. In such data-rich environments, it becomes not only feasible but also valuable and necessary to detect DIF in highly granular subgroup structures, such as those arising from intersectionality, where multiple demographic or contextual variables interact to create numerous small subgroups. These challenges are especially prominent in large-scale assessments and high-stakes testing contexts, including university admissions, workforce certification exams, and psychological evaluations. In such settings, fairness across subpopulations is a critical concern, and the consequences of unaddressed measurement bias can be severe. Traditional DIF detection methods often struggle under these conditions due to limited subgroup sizes and methodological asymmetries, such as the need to prespecify a reference group. The proposed method addresses these limitations by enabling flexible, symmetric comparisons among all group pairs, thereby improving the detection and correction of potential biases. In this way, our approach supports ongoing efforts to enhance equity and accountability in assessment practices. Its adoption can inform more inclusive and representative test development, contribute to fairer outcomes for examinees, and help align measurement practices with broader societal goals related to justice, diversity, and inclusion. As testing programs increasingly seek to serve heterogeneous populations, the ability to detect subtle and complex forms of DIF will be critical to ensuring that assessments remain defensible and ethically responsible.

To demonstrate the practical utility of the proposed method, we apply it to two real-world datasets and find that, despite their long-standing and widespread use, these assessments continue to exhibit notable DIF. However, detecting DIF is not the final goal; rather, it constitutes a crucial first step in the broader process of building equitable assessments. Ultimately, psychometricians should collaborate with subject-matter experts to interpret the results, and to review, revise, or remove flagged items as appropriate. Such interdisciplinary collaboration is key to promoting fairness, reducing bias, and enhancing the interpretability, credibility, and validity of test scores across diverse populations.

Although the proposed method demonstrates superior performance in terms of high true positive rates and low false positive rates in simulation studies, it also presents several limitations that suggest directions for future research. First, the method requires the specification of two tuning parameters: 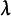 , which controls the overall strength of the penalty, and 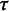 , which determines the truncation threshold in the TLP. Fine-tuning these parameters via a two-dimensional grid search is computationally intensive, particularly in large-scale applications. Future work could explore more efficient tuning strategies to alleviate this computational burden. Second, model estimation is carried out using the EM algorithm in conjunction with Gaussian quadrature. While effective, this approach can be computationally demanding and is only practical for models with low-dimensional latent traits. A promising alternative is the use of Gaussian variational estimation methods, which have demonstrated strong performance in high-dimensional settings (Cho et al., 2021; Lyu et al., 2025; Ma et al., 2024). Adopting such approaches could significantly improve scalability and broaden the method’s applicability to more complex testing scenarios. Third, while the current approach is designed for the 2PL model, extending the approach to accommodate other IRT models, such as the graded response model or the partial credit model, would enhance its utility for polytomous items. Similarly, applications to assessments containing items of mixed formats are increasingly relevant and warrant further investigation. Finally, although this study employs the TLP to address the bias introduced by the LP, several alternative debiasing strategies exist. These include the adaptive lasso (Schauberger & Mair, 2020; Wang et al., 2023; Zou, 2006), the MCP (Belzak, 2023; Zhang, 2010), and the smoothly clipped absolute deviation (SCAD) penalty (Fan & Li, 2001). A comprehensive empirical comparison of these regularization techniques, considering both computational efficiency and statistical accuracy, would provide valuable guidance for methodologists and practitioners working on DIF detection.

## Footnotes

1 We also explored an alternative approach by flagging an item as DIF between two groups if DIF was detected when either group served as the reference. However, this strategy resulted in too high false positive rates for both IW-GVEMM and MCP.
